# Detectable HBV DNA during nucleos(t)ide analogues stratifies predictive hepatocellular carcinoma risk score

**DOI:** 10.1038/s41598-020-69522-w

**Published:** 2020-08-03

**Authors:** Shun Kaneko, Masayuki Kurosaki, Kouji Joko, Hiroyuki Marusawa, Masahiko Kondo, Yuji Kojima, Yasushi Uchida, Hiroyuki Kimura, Keiji Tsuji, Hitoshi Yagisawa, Atsunori Kusakabe, Haruhiko Kobashi, Takehiro Akahane, Nobuharu Tamaki, Sakura Kirino, Takehiko Abe, Hideo Yoshida, Tomomichi Matsushita, Chitomi Hasebe, Namiki Izumi

**Affiliations:** 1 Department of Gastroenterology and Hepatology, Musashino Red Cross Hospital, 1-26-1, Kyonan-cho, Musashino-shi, Tokyo, 180-8610 Japan; 2 Center for Liver-Biliary-Pancreatic Diseases, Matsuyama Red Cross Hospital, Ehime, Japan; 3 Department of Gastroenterology, Japanese Red Cross Osaka Hospital, Osaka, Japan; 4 Department of Gastroenterology, Japanese Red Cross Otsu Hospital, Shiga, Japan; 5 Department of Gastroenterology, Japanese Red Cross Ise Hospital, Mie, Japan; 6 Department of Gastroenterology, Japanese Red Cross Matsue Hospital, Shimane, Japan; 7 Department of Gastroenterology, Japanese Red Cross Kyoto Daiichi Hospital, Kyoto, Japan; 8 Department of Gastroenterology, Hiroshima Red Cross Hospital and Atomic-Bomb Survivors Hospital, Hiroshima, Japan; 9 Department of Gastroenterology, Japanese Red Cross Akita Hospital, Akita, Japan; 10 Department of Gastroenterology, Japanese Red Cross Nagoya Daini Hospital, Nagoya, Japan; 11 Department of Hepatology, Japanese Red Cross Okayama Hospital, Okayama, Japan; 12 Department of Gastroenterology, Japanese Red Cross Ishinomaki Hospital, Miyagi, Japan; 13 Department of Gastroenterology, Japanese Red Cross Maebashi Hospital, Gunma, Japan; 14 Department of Gastroenterology, Japanese Red Cross Medical Center, Tokyo, Japan; 15 Department of Gastroenterology, Japanese Red Cross Gifu Hospital, Gifu, Japan; 16 Department of Gastroenterology, Japanese Red Cross Asahikawa Hospital, Hokkaido, Japan

**Keywords:** Hepatitis, Hepatology, Liver cancer, Risk factors

## Abstract

Nucleos(t)ide analogs (NA) suppress hepatitis B virus (HBV) replication and reduce the risk of hepatocellular carcinoma (HCC). However, NA cannot suppress carcinogenesis completely in patients with chronic hepatitis B. The aims of this study were to identify risk factors for HCC and develop a refined carcinogenesis prediction model. Patients receiving NA therapy (n = 1,183) were recruited retrospectively from the 16 hospitals. All patients had been receiving NA continuously for more than 1 year until the end of the follow-up. During a median follow-up of 4.9 (1.0–12.9) years, 52 (4.4%) patients developed HCC. A multivariate analysis revealed that male gender, older age, lower platelet counts at the baseline, and detectable HBV DNA during NA therapy were independent predictive factors of HCC development. The PAGE-B score was calculated by using these factors. 240 (20.3%), 661 (55.9%), and 282 (23.8%) patients were classified into low-, intermediate-, and high-risk groups, respectively. In the intermediate- and high-risk group, detectable HBV DNA was significantly associated with a higher risk of HCC development compared with continuously undetectable HBV DNA, respectively (HR 3.338; 95% CI 1.045–10.66/HR 3.191; 95% CI 1.543–6.597). PAGE-B–DNA, which is the combined PAGE-B and HBV DNA status, was valuable for a more refined stratification of PAGE-B.

## Introduction

Hepatitis B virus (HBV) infection remains a major health threat. 257 million people worldwide are infected with HBV, which involves a risk of cirrhosis and hepatocellular carcinoma (HCC). This infection is responsible for more than 887,000 deaths annually^1^. Nucleos(t)ide analogs (NA) suppress HBV replication and reduce the risk of HCC and HBV-associated mortality^2–7^. Reports of NA therapy pertained mainly to entecavir (ETV), whereas the number of studies of tenofovir disoproxil fumarate (TDF) has increased in recent years^8–11^. Recently, tenofovir alafenamide (TAF), which was designed to exhibit greater plasma stability compared with TDF, was approved for clinical application. TAF is as effective as TDF and leads to continuous improvement in renal and bone safety in the treatment of patients with chronic hepatitis B (CHB)^12–15^. Reports of the reduction of the risk of HCC development induced by TAF are scarce, because of their short observation periods. NA cannot eliminate HBV from the host cells because of the persistence of HBV covalently closed circular DNA, which serves as the template for viral transcription^16,17^. Therefore, long-term treatment is necessary for HBV suppression.

As described above, there is some evidence of the reduction of the risk of HCC after treatment with NA; however, the authors did not mention the complete suppression of HCC. Several studies have addressed the risk of CHB patients received NA. Most reports pertain to age, gender, and platelet counts^18–20^. The PAGE-B score was generated based on these factors^21,22^. The PAGE-B score exhibits a high versatility and has been applied extensively worldwide. However, the number of patients in the intermediate PAGE-B group tends to be elevated. Recently, several studies reported that complete viral suppression reduced the risk of developing HCC^23–25^. Conversely, even low detectable levels of HBV DNA in the serum confer a risk of HCC development. The interaction between the host and the virus is very important for HBV pathogenesis. Therefore, we attempted to construct a prediction HCC model in CHB patients receiving NA therapy using a well-designed representative risk score of the host (PAGE-B) or of the virus (HBV DNA status).

The aims of this study were to develop a refined carcinogenesis prediction model based on conventional prediction models (PAGE-B and serum HBV DNA) in patients receiving NA therapy. Previously, we conducted a nationwide HBV cohort study^26^, which was developed further here. The risk factors for HCC among CHB patients were investigated via a multicenter (Japanese Red Cross Liver Study Group) analysis. Moreover, we assessed that the HBV DNA status on NA therapy discriminated the PAGE-B intermediate-risk group and the high-risk group.

## Results

### Baseline patient characteristics

All CHB patients received NA therapy continuously. Between October 2006 and August 2018, 1652 patients started to receive NA therapy (ETV/TDF/TAF = 1,010/270/372). Patients who were observed for less than 1 year, had a past HCC history, or exhibited HCC development within the first year of treatment were not included in this cohort. In total, 1,183 patients on NA therapy were included in the analyses. There were 700 naïve-treatment patients and 483 patients had prior history of treatment. The total cumulative number of patients previously treated by NA other than ETV/TDF/TAF were 381, and by interferon-based therapy 169. Among them, 703 (59.4%) patients were male; the mean age was 52.9 ± 12.9 years; 177 (14.9%) patients were diagnosed with cirrhosis; and 401 (33.8%) patients were hepatitis B e antigen (HBeAg) positive. The baseline serum data (platelet, albumin, bilirubin, alanine aminotransferase, alpha fetoprotein, creatinine, and serum HBV DNA levels) are described in Table 1. The median follow-up duration was 4.9 (1.0–12.9) years. Finally, 780 (65.9%), 206 (17.4%), and 197 (16.7%) patients received ETV, TDF, and TAF therapy, respectively.

**Table 1:** Clinical characteristics of NA-treated patients at baseline.

	n = 1,183
Male gender (n, %)	703 (59.4%)
Age (years)	52.9 ± 12.9
Cirrhosis (n, %)	177 (14.9%)
Diabetes mellitus (n, %)	121 (10.2%)
Platelet (× 10^9^/l)	182 ± 92
Albumin (g/dl)	4.2 ± 0.5
Total bilirubin (mg/dl)	0.9 ± 0.9
Alanine aminotransferase (IU/l)	114.4 ± 247.6
Alpha fetoprotein (ng/ml)	14.5 ± 59.4
Creatinine (mg/dl)	0.8 ± 0.6
HBeAg positivity (n, %)	401 (33.8%)
HBV DNA level (IU/ml) (n, %)	
< 2000	403 (34%)
2000–200,000	195 (16.5%)
> 200,000	585 (49.5%)
Follow-up duration (years)	4.9 (1.0–12.9)
Antiviral therapy (n, %)	
Entecavir	780 (65.9%)
Tenofovir disoproxil fumarate	206 (17.4%)
Tenofovir alafenamide	197 (16.7%)

*NA* nucleos(t)ide analogues, *HBV DNA* hepatitis B virus deoxyribonucleic acid, *HBeAg* HBV e antigen.

### Cumulative incidence of HCC

**Figure 1:**
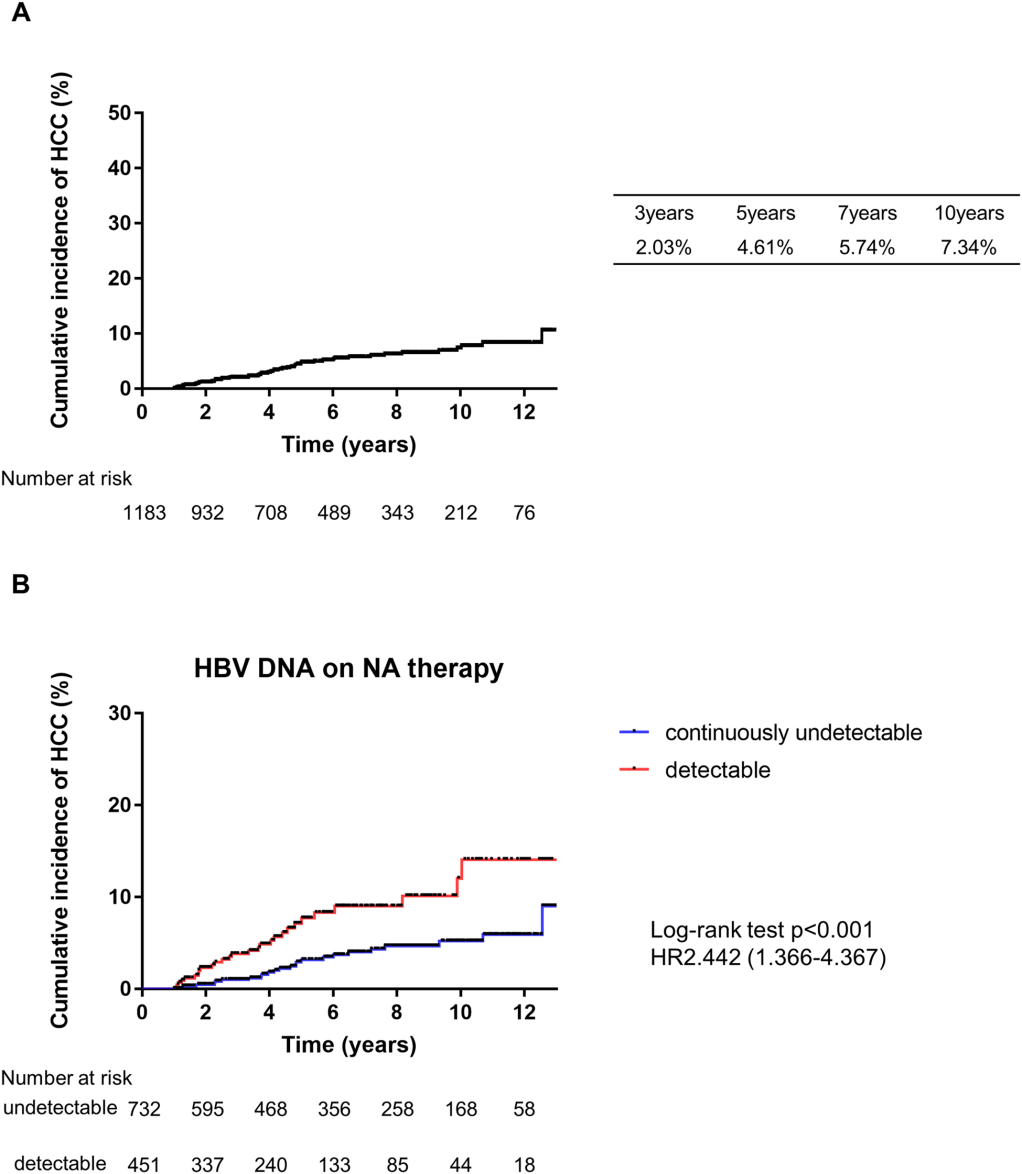
Cumulative incidence of hepatocellular carcinoma. (**A**) All CHB patients received NA therapy. (**B**) HBV DNA status; 732 (61.8%) and 451 (38.2%) patients achieved continuously undetectable HBV DNA and detectable HBV DNA status, respectively. Patients who did or did not achieve continuously undetectable HBV DNA status (log-rank test, *P* < 0.001).

During the follow-up, 52 (4.39%) patients developed HCC as shown clinical characteristics at diagnosis in Table 2. The cumulative incidence rates of HCC at 3, 5, 7, and 10 years after ETV/TDF/TAF treatment were 2.03%, 4.61%, 5.74%, and 7.34%, respectively (Fig. 1A). There were 27 patients who died by almost non-liver related causes (other cancer 15, intracranial hemorrhage 1, pneumonia 2, heart failure 1, renal failure 1, liver related death 3, unknown 4).

**Table 2:** Clinical characteristics of HCC patients at diagnosis.

	n = 52
Male gender (n, %)	43 (82.7%)
Age (years)	64.6 ± 9.5
Cirrhosis (n, %)	27 (51.9%)
Alanine aminotransferase (IU/l)	28.5 ± 21.7
Albumin (g/dl)	4.2 ± 0.4
Total bilirubin (mg/dl)	1.0 ± 0.6
BCLC stage 0/A/B/C/D (n)	26/21/3/2/0
Treatment modalities (n) RFA/resection/TACE/sorafenib	37/9/5/1
HBV DNA status (n) Continuously undetectable/detectable	23/29

*HCC* hepatocellular carcinoma, *BCLC* Barcelona Clinic Liver Cancer, *RFA* radiofrequency ablation, *TACE* transarterial chemoembolization, *HBV DNA* hepatitis B virus deoxyribonucleic acid.

### Factors associated with HCC development during NA therapy

In addition to the baseline characteristics that were used for the analysis of factors associated with HCC, we also focused on the HBV DNA status during NA therapy. We investigated the effects of these HBV DNA status in CHB patients on liver carcinogenesis. We found that 732 (61.8%) and 451 (38.2%) patients achieved continuously undetectable and detectable HBV DNA status, respectively. The detectable HBV DNA was significantly associated with a higher risk of HCC development compared with the continuously undetectable HBV DNA (HR 2.442; 95% CI 1.366–4.367; log-rank test, *P* < 0.001; Fig. 1B). For detail, the median serum HBV DNA in patients with detectable DNA were 2.5 (1.88, 3.25) Log IU/ml. The virological breakthrough was seen in 2 patients whose HBV resistant mutants were found. They all were rescued by adding TDF to ETV. There were 36 patients who never achieved undetectable HBV DNA and 141 patients who had virological relapse after complete suppression, respectively. However, these factors were not significantly different in the risk of HCC development.

27 patients achieved hepatitis B surface antigen (HBsAg) seroclearance and no patient developed HCC.

Next, we investigated the predictive factors of HCC using univariate and multivariate analysis. In the univariate analysis, male gender, older age, cirrhosis, lower platelet counts (< 150 × 10^9^/l), lower albumin levels, higher bilirubin levels, the ALBI^27^ score (which is consisted albumin and bilirubin and useful for the evaluation of liver function), alcohol drinking, and detectable HBV DNA were significant predictive factors of HCC. In the multivariate analysis, male gender (HR 3.731; 95% CI 1.669–8.340; *P* = 0.001), older age (HR 1.06; 95% CI 1.033–1.088; *P* < 0.001), cirrhosis (HR 2.59; 95% CI 1.288–5.205; *P* = 0.007), lower platelet counts (HR 2.763; 95% CI 1.231–6.203; *P* = 0.013), and detectable HBV DNA (HR 3.234; 95% CI 1.732–6.035; *P* < 0.001) were independent predictive factors of HCC development during NA therapy (Table 3).

**Table 3:** Cox proportional hazards regression analysis for factors associated with HCC.

	Univariate analysis			Multivariate analysis		
	HR	95%CI	*P* value	HR	95%CI	*P* value
Male gender	3.362	1.639–6.898	< 0.001	3.731	1.669–8.340	0.001
Age	1.068	1.038–1.085	< 0.001	1.06	1.033–1.088	< 0.001
Cirrhosis	5.334	3.092–9.201	< 0.001	2.59	1.288–5.205	0.007
Diabetes mellitus	1.164	0.494–2.744	0.729			
Platelet (< 150 × 10^9^/l)	4.468	2.419–8.258	< 0.001	2.763	1.231–6.203	0.013
Albumin	0.498	0.315–0.788	0.003	0.191	0.115–3.148	0.247
Total bilirubin	1.125	1.008–1.255	0.036	0.868	0.646–1.167	0.348
ALBI score	2.416	1.517–3.850	0.002	8.748	0.367–208.5	0.18
Alanine aminotransferase	0.999	0.998–1.001	0.455			
Creatinine	1.208	0.787–1.855	0.386			
Positive HBeAg	0.779	0.436–1.392	0.399			
Antiviral therapy ETV (vs. TDF/TAF)	0.578	0.201–1.660	0.308			
Alcohol drinking	3.058	1.550–6.031	0.001	2.141	0.989–4.464	0.061
Detectable HBV DNA during NA therapy	2.475	1.429–4.286	0.001	3.234	1.732–6.035	< 0.001

*HR* hazards ratios, *HCC* hepatocellular carcinoma, *ALBI* Albumin-Bilirubin, *HBeAg* hepatitis B virus e antigen, *ETV* Entecavir, *TDF* Tenofovir disoproxil fumarate, *TAF* Tenofovir alafenamide, *HBV DNA* hepatitis B virus deoxyribonucleic acid, *NA* nucleos(t)ide analogues.

Furthermore, we performed a subgroup analysis with the HBV DNA status of cirrhosis and HBeAg. HBV DNA status during nucleos(t)ide analogues could significantly stratify the risk of HCC development in these subgroups (Supplementary Figure 1).

Particularly in treatment naive patients (n = 700), the cumulative incidence rate of HCC in patients who had higher pretreatment HBV DNA levels (> 4.0 log IU/ml) was significantly higher (HR 5.446; 95% CI 2.111–14.05; log-rank test, *P* = 0.0413) (Supplementary Figure 2A). As a predict factor for achievement continuously undetectable HBV DNA, serum HBV DNA 1 year after starting NA significantly related achievement continuously undetectable HBV DNA (*P* < 0.0001, Logistic regression). HBV DNA status 1 year after starting NA was also the significant risk factor of HCC development (HR 2.279; 95% CI 1.214–4.278; log-rank test, *P* = 0.0303) (Supplementary Figure 2B).

### HBV DNA status stratifies the PAGE-B HCC predictive score

**Figure 2:**
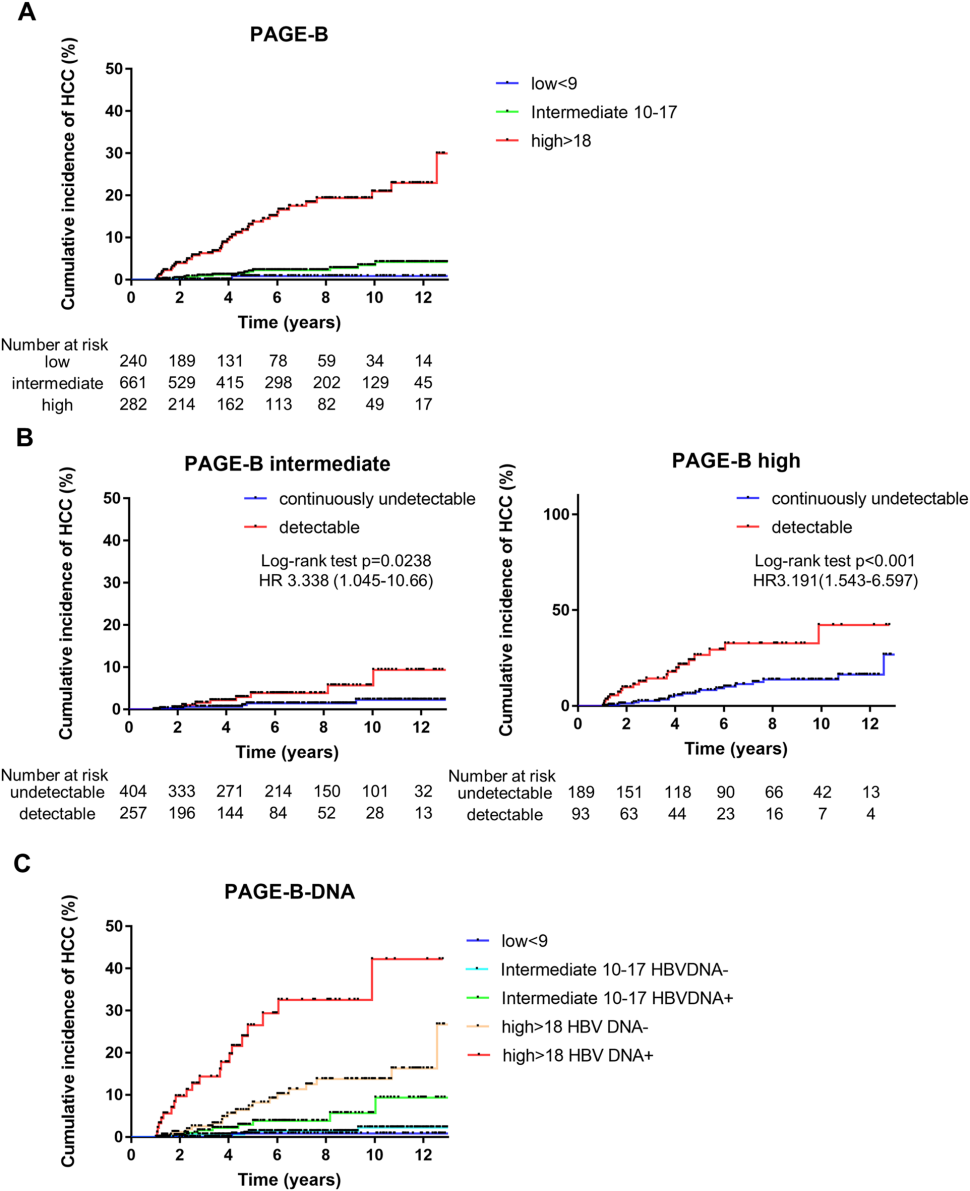
Cumulative incidence of hepatocellular carcinoma with PAGE-B score subdivided according to HBV DNA status on NA therapy. (**A**) Cumulative incidence rates of HCC based on PAGE-B. The PAGE-B score classified 240 (20.3%), 661 (55.9%), and 282 (23.8%) patients into the low-, intermediate-, and high-risk groups, respectively. (**B**) Patients who did or did not achieve a continuously undetectable HBV DNA status in intermediate- and high-risk PAGE-B groups. HBV DNA status classified the intermediate- and high-risk patients (based on PAGE-B) into subgroups (log-rank test, *P* = 0.0238, *P* < 0.001). (**C**) The PAGE-B score was stratified using the HBV DNA status.

As described above, male gender, older age, and lower platelet counts were independent predictive factors of HCC development during NA therapy. The PAGE-B score was developed using these factors in CHB patients on antiviral therapy and exhibited a high concordance index value for the prediction of HCC development^21,22^. We validated the PAGE-B score in our cohort (Fig. 2A) and found that it was sufficiently useful.

We classified 240 (20.3%), 661 (55.9%), and 282 (23.8%) patients into low-, intermediate-, and high-risk groups, respectively. However, the majority of patients were in the intermediate-risk group. Therefore, we investigated whether HBV DNA status on NA therapy could subdivide the PAGE-B intermediate-risk group.

We observed that 404 (61.1%) and 257 (38.9%) patients achieved a continuously undetectable and a detectable HBV DNA, respectively. In the PAGE-B intermediate-risk group, the detectable HBV DNA was significantly associated with a higher risk of HCC development compared with the continuously undetectable HBV DNA (HR 3.338; 95% CI 1.045–10.66; log-rank test, *P* = 0.0238; Fig. 2B, left panel). Furthermore, in the PAGE-B high-risk group, the detectable HBV DNA was also significantly associated with a higher risk of HCC development (HR 3.191; 95% CI 1.543–6.597; log-rank test, *P* < 0.001; Fig. 2B, right panel). The cumulative incidence rate of HCC in the PAGE-B high-risk group with a continuously negative HBV DNA was significantly higher compared with that observed in the PAGE-B intermediate-risk with a detectable HBV DNA status (HR 2.369; 95% CI 1.098–5.111; log-rank test, *P* = 0.0352). A single case of HCC development was detected in the PAGE-B low-risk group. Thus, the low-risk group could show function as it was.

Based on these results, PAGE-B was significantly stratified when divided into five groups based on HBV DNA status (Fig. 2C). HBV DNA status on NA therapy was complementary to the PAGE-B score.

This PAGE-B–DNA prediction model is useful for the rigorous assessment of the cumulative incidence rate of HCC at 3, 5, 7, and 10 years in CHB patients who are on antiviral therapy (Table 4). The discriminative ability of our models was compared by Akaike's Information Criterion (AIC). AIC values were so small that better discriminative ability of predicting HCC development was shown in PAGE-B-DNA (Supplementary Table 1).

**Table 4:** Cumulative incidence rate of HCC with PAGE-B-DNA prediction model stratifications at 3,5,7 and 10 years.

PAGE-B-DNA (n = 1,183)	n, %	3 years	5 years	7 years	10 years
low (< 9)	240 (20.3%)	0%	0.81%	0.81%	0.81%
intermediate (10–17) HBV DNA−	404 (34.2%)	0.59%	1.39%	1.39%	2.27%
intermediate (10–17) HBV DNA+	257 (21.7%)	1.56%	2.89%	3.82%	5.71%
high (> 18) HBV DNA−	189 (16.0%)	2.53%	8.21%	11.30%	13.73%
high (> 18) HBV DNA+	93 (7.8%)	14.32%	26.52%	32.56%	42.19%

*HCC* hepatocellular carcinoma; *PAGE-B* platelets, age, gender-hepatitis B scores; *HBV DNA* hepatitis B virus deoxyribonucleic acid.

## Discussion

This study provides the first evidence that HBV DNA status on NA therapy is useful for subdividing further the PAGE-B score. NA therapy suppress the risk of HCC and liver-related death. However, it did not mean that NA therapy in CHB patients suppressed HCC completely^3^. A report showed that surveillance leads to early detection of HCC and suppresses cancer-related death in patients with HBV^28^. Therefore, a simple and appreciate study aimed at evaluating the risk factors of HCC development during NA therapy is needed.

In our cohort, the cumulative incidence rates of HCC were 4.61% at 5 years and 7.34% at 10 years, which was in agreement with previous reports^3–7^ (Fig. 1A). Several previous studies addressed the HBV DNA status^23–25^. Here, male gender, old age, cirrhosis, lower platelet counts at the baseline, and HBV DNA during NA therapy were validated as significant factors of liver carcinogenesis in CHB patients (Fig. 1B, Table 3).

Many risk scores have been reported (CU-HCC, GAG-HCC, REACH-B, PAGE-B, mPAGE-B, etc.)^23,29–32^. Of them, PAGE-B was the most convenient score because of its high level of versatility^21^. As depicted in Fig. 2A, risk stratification was possible using PAGE-B. However, the number of intermediate-risk cases was particularly large, and the rate of cumulative incidence of the high-risk group was far from that of the intermediate-risk group. Therefore, subclassification was performed in these two groups. HBV DNA status significantly stratified the risk of HCC in both risk groups (Fig. 2B). Interestingly, the cumulative incidence rate of HCC in the PAGE-B high-risk group with a continuously negative HBV DNA status was significantly higher compared with the PAGE-B intermediate-risk group with a detectable HBV DNA status. It was suggested that the PAGE-B score was the main classifier, with HBV DNA status on NA therapy working complementary. These results showed that a more detailed risk assessment was possible (Fig. 2C, Table 4, Supplementary Table 1). This “PAGE-B–DNA” system was constructed using very simple parameters (age, gender, platelet counts, and HBV DNA) that can be measured during the clinical follow-up. This risk assessment exhibited a greater stratification and was highly useful. Moreover, the modified PAGE-B score, which was constructed by adding albumin level to the PAGE-B factors^32^, was also supported by subdivision using this complementary HBV DNA status on NA therapy (Supplementary Figure 3, Supplementary Tables 1, 2).

This study was valuable because it included the host factor together with a viral factor, i.e., HBV DNA status, during therapy. The intervention to viral factors may be possible, though intervention to host factors is difficult. Several approaches (switching NA, interferon sequential therapy, new therapeutic agents, etc.) are considered to achieve continuously undetectable HBV DNA. The further interventions to virus might yield better results for CHB patients during NA therapy. A few articles reported the risk factors for HCC development during NA therapy in Japan^26,33–35^. Therefore, such a Japanese nationwide multicenter analysis was very valuable.

However, this study had several limitations. First, this was a retrospective study in which the exclusion of unidentified biases was impossible. Second, regarding HBV DNA status on NA therapy, we were unable to determine why the HBV DNA was detectable. If possible, virus resistance mutations and patients’ adherence to medication should be examined. Lack of data and serum samples precluded the performance of such analyzes here. Several characteristics were available for the CHB patients included in this cohort; i.e., this study was based on real-world data and seem to be useful for actual clinical practice. External validation is needed for implementing prediction models in clinical practice. Therefore, further study including the external validation cohort is expected.

In conclusion, HBV DNA status was useful for stratifying HCC risk in CHB patients on NA therapy. Furthermore, the PAGE-B-DNA system, which combined PAGE-B with HBV DNA status, was valuable because it provided a more detailed stratification compared with PAGE-B alone. The application of PAGE-B-DNA to appreciate surveillance may lead to the early detection and treatment of HCC, thus helping to improve the prognosis of CHB patients.

## Method

### Patients

Patients receiving NA therapy (n = 1652) were recruited retrospectively from the 16 hospitals that are part of the Japanese Red Cross Liver Study Group. All patients were HBsAg positive for more than 6 months and serum HBV DNA positive before NA therapy. No patients had coinfection with the hepatitis C virus or human immunodeficiency virus.

469 patients were excluded because of a short follow-up duration (less than 1 year) or the development of HCC before NA therapy or within 1 year of NA therapy onset. Finally, 1,183 patients were included in the analyses.

All patients received NA therapy continuously for more than 1 year until the end of the follow-up. The study was approved by the institutional review board of each hospital (e.g. Musashino Red Cross Hospital Ethics Review Committee), in accordance with the Declaration of Helsinki. The written informed consent was obtained from all patients.

### Clinical evaluation and follow-up

The age and gender of the patients were recorded at the time of study entry. HBeAg and anti-HBe antibodies (HBeAb) were determined using commercially available enzyme immunoassay kits at each hospital. The quantitative measurement of HBV DNA and HBsAg was performed using real-time PCR (COBAS 6800/8800 system, TaqMan HBV assay; Roche) and a Chemiluminescence Immunoassay (CLIA; Abbott Japan), respectively. Cirrhosis was assessed based on the presence of clinical, radiological, endoscopic, and laboratory evidence (platelet count, < 100 × 10^9^/l, and a blunted, nodular liver edge accompanied by splenomegaly > 12 cm) or clinical symptoms of portal hypertension, such as ascites, esophageal or gastric varices, and hepatic encephalopathy.

Ultrasonography and blood tests, including assays of tumor markers, were performed every 3–6 months for HCC surveillance. When tumor marker levels rose abnormally and/or abdominal ultrasonography suggested a lesion that was suspicious for HCC, contrast-enhanced computed tomography, magnetic resonance imaging, or angiography was performed. HCC was diagnosed in tumors that displayed vascular enhancement at the early phase and washout at the later phase, according to the guidelines of the American Association for the Study of Liver Diseases^36^, the European Association for the Study of the Liver^37^, and the Japan Society of Hepatology^38^. Tumor biopsy was used to support diagnoses. The primary outcome was HCC development in CHB patients on NA therapy.

### HBV DNA status

In many cases, NA treatment causes HBV DNA negative. There are two possible clinical courses. Therefore, we defined HBV DNA status on NA therapy as described below.

#### Continuously undetectable HBV DNA

The serum HBV DNA was quantified by real-time PCR (using the COBAS 6800/8800 system mainly) with a detection range of 1.0–9.0 Log IU/ml. The lower limit was 10 IU/ml. A continuously undetectable status was defined as an undetectable level of serum HBV DNA that was maintained up to the last observation, excluded detectable under 10 IU/ml on NA therapy.

#### Detectable HBV DNA

A detectable status was defined as a literally detectable serum HBV DNA and included occasionally detected HBV DNA among undetectable cases during the follow-up.

### Statistical analyzes

Cumulative incidences of HCC development curves were prepared using the Kaplan–Meier method. The cumulative incidence curves were compared using the log-rank Mantel–Cox test. The factors associated with HCC development were analyzed using a Cox proportional hazards model. Significance was set at *P* < 0.05. The GraphPad Prism software (GraphPad Software, San Diego, CA, USA) and EZR (Saitama Medical Center, Jichi Medical University, Shimotsuke, Japan) were used to analyze statistical significance.

## Supplementary information

Supplementary Tables.

Supplementary Figure Legends.

Supplementary Figure 1.

Supplementary Figure 2.

Supplementary Figure 3.
