# Supplementary material for: Detectable HBV DNA during nucleos(t)ide analogues stratifies predictive hepatocellular carcinoma risk score
Source: Sci Rep. 2020 Aug 3;10:13021. doi: 10.1038/s41598-020-69522-w (PMC7400741; doi:10.1038/s41598-020-69522-w)
Supplement: Supplementary file 1 — Supplementary Tables. [file 41598_2020_69522_MOESM1_ESM.docx]

**Detectable HBV DNA during nucleos(t)ide analogues stratifies predictive hepatocellular carcinoma risk score**

Shun Kaneko^1^, Masayuki Kurosaki^1^, Kouji Joko^2^, Hiroyuki Marusawa^3^, Masahiko Kondo^4^, Yuji Kojima^5^, Yasushi Uchida^6^, Hiroyuki Kimura^7^, Keiji Tsuji^8^, Hitoshi Yagisawa^9^, Atsunori Kusakabe^10^, Haruhiko Kobashi^11^, Takehiro Akahane^12^, Nobuharu Tamaki^1^, Sakura Kirino^1^, Takehiko Abe^13^, Hideo Yoshida^14^, Tomomichi Matsushita^15^, Chitomi Hasebe^16^, Namiki Izumi^1^

^1^Department of Gastroenterology and Hepatology, Musashino Red Cross Hospital, ^2^Center for Liver-Biliary-Pancreatic Diseases, Matsuyama Red Cross Hospital, ^3^Department of Gastroenterology, Japanese Red Cross Osaka Hospital, ^4^Department of Gastroenterology, Japanese Red Cross Otsu Hospital, ^5^Department of Gastroenterology, Japanese Red Cross Ise Hospital, ^6^Department of Gastroenterology, Japanese Red Cross Matsue Hospital, ^7^Department of Gastroenterology, Japanese Red Cross Kyoto Daiichi Hospital, ^8^Department of Gastroenterology, Hiroshima Red Cross Hospital & Atomic-bomb Survivors Hospital, ^9^Department of Gastroenterology, Japanese Red Cross Akita Hospital, ^10^Department of Gastroenterology, Japanese Red Cross Nagoya Daini Hospital, ^11^Department of Hepatology, Japanese Red Cross Okayama Hospital, ^12^Department of Gastroenterology, Japanese Red Cross Ishinomaki Hospital, ^13^Department of Gastroenterology, Japanese Red Cross Maebashi Hospital, ^14^Department of Gastroenterology, Japanese Red Cross Medical Center, ^15^Department of Gastroenterology, Japanese Red Cross Gifu Hospital, ^16^Department of Gastroenterology, Japanese Red Cross Asahikawa Hospital, Japan.

Correspondence to: Namiki Izumi, M.D., Ph.D.,

Department of Gastroenterology and Hepatology, Musashino Red Cross Hospital

1-26-1, Kyonan-cho, Musashino-shi Tokyo 180-8610, Japan.

E-mail: izumi012@musashino.jrc.or.jp (N.Izumi)

**Supplementary Table.1 The Akaike's Information Criterion values of existing models and newly proposed models.**

|  | **PAGE-B** | **mPAGE-B** |
| --- | --- | --- |
| all (n=1183) | 670.69 | 629.63 |
|  | **PAGE-B-DNA** | **mPAGE-B-DNA** |
| detectable HBV DNA (n=451) | 306.23 | 285.8 |
| continuously undetectable HBV DNA(n=732) | 284.01 | 270.02 |

mPAGE-B, modified platelets, age, gender-hepatitis B scores; HBV DNA, hepatitis B virus deoxyribonucleic acid.

**Supplementary Table.2 Cumulative incidence rate of HCC with modified PAGE-B-DNA prediction model stratifications at 3,5,7 and 10 years.**

| mPAGE-B-DNA (n=1141) | n, % | 3years | 5years | 7years | 10years |
| --- | --- | --- | --- | --- | --- |
| low (<8) | 277 (24.2%) | 0% | 0% | 0% | 0% |
| intermediate (9-12) HBV DNA- | 290 (25.5%) | 0.43% | 1.51% | 1.52% | 1.52% |
| intermediate (9-12) HBV DNA+ | 186 (16.3%) | 1.41% | 3.03% | 5.62% | 5.62% |
| high (>13) HBV DNA- | 261 (22.9%) | 2.26% | 7.15% | 8.62% | 12.09% |
| high (>13) HBV DNA+ | 127 (11.1%) | 11.76% | 20.87% | 20.87% | 26.52% |

HCC, hepatocellular carcinoma; mPAGE-B, modified platelets, age, gender-hepatitis B scores; HBV DNA, hepatitis B virus deoxyribonucleic acid.
