# Supplementary material for: Detectable HBV DNA during nucleos(t)ide analogues stratifies predictive hepatocellular carcinoma risk score
Source: Sci Rep. 2020 Aug 3;10:13021. doi: 10.1038/s41598-020-69522-w (PMC7400741; doi:10.1038/s41598-020-69522-w)
Supplement: Supplementary file 2 — Supplementary Figure Legends. [file 41598_2020_69522_MOESM2_ESM.docx]

**Supplemental Figure 1. Cumulative incidence of hepatocellular carcinoma with HBV DNA status sub-grouped by HBeAg status and cirrhosis**

**(A) HBeAg positive and negative patients.**

**(B)Chronic hepatitis patients (CH) and Liver cirrhosis patients (LC)**

**Supplemental Figure 2. Cumulative incidence of hepatocellular carcinoma of treatment naïve patients with chronic hepatitis B**

**(A) Serum HBV DNA levels before antiviral therapy**

**(B) Serum HBV DNA status 1 year after antiviral therapy**

**Supplemental Figure 3. Cumulative incidence of hepatocellular carcinoma with modified PAGE-B score subdivided according to HBV DNA status on NA therapy**
