# Supplementary figures and images for: Detectable HBV DNA during nucleos(t)ide analogues stratifies predictive hepatocellular carcinoma risk score
Source: Sci Rep. 2020 Aug 3;10:13021. doi: 10.1038/s41598-020-69522-w (PMC7400741; doi:10.1038/s41598-020-69522-w)

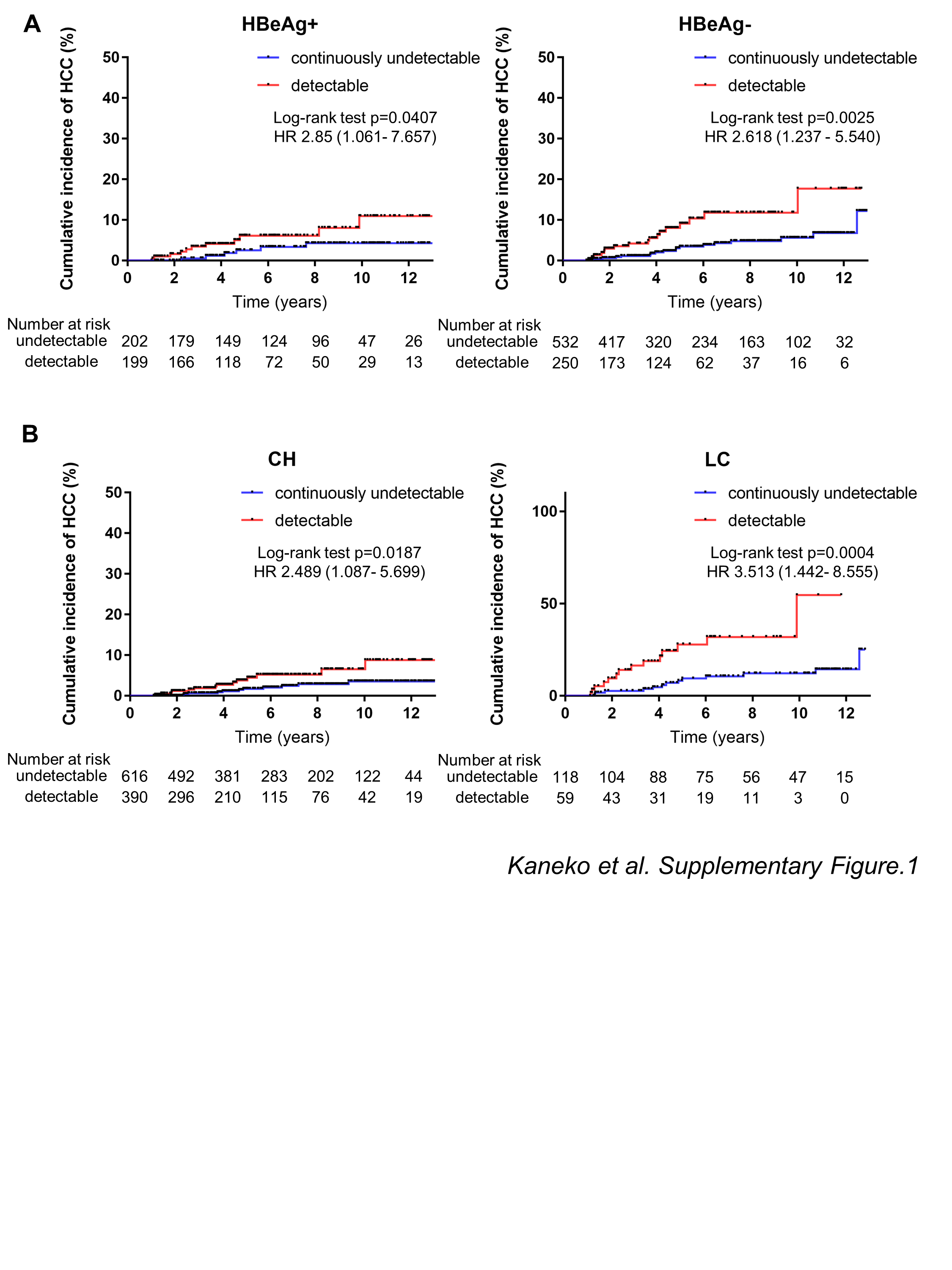

Supplement: Supplementary file 3 — Supplementary Figure 1. [file 41598_2020_69522_MOESM3_ESM.tif]

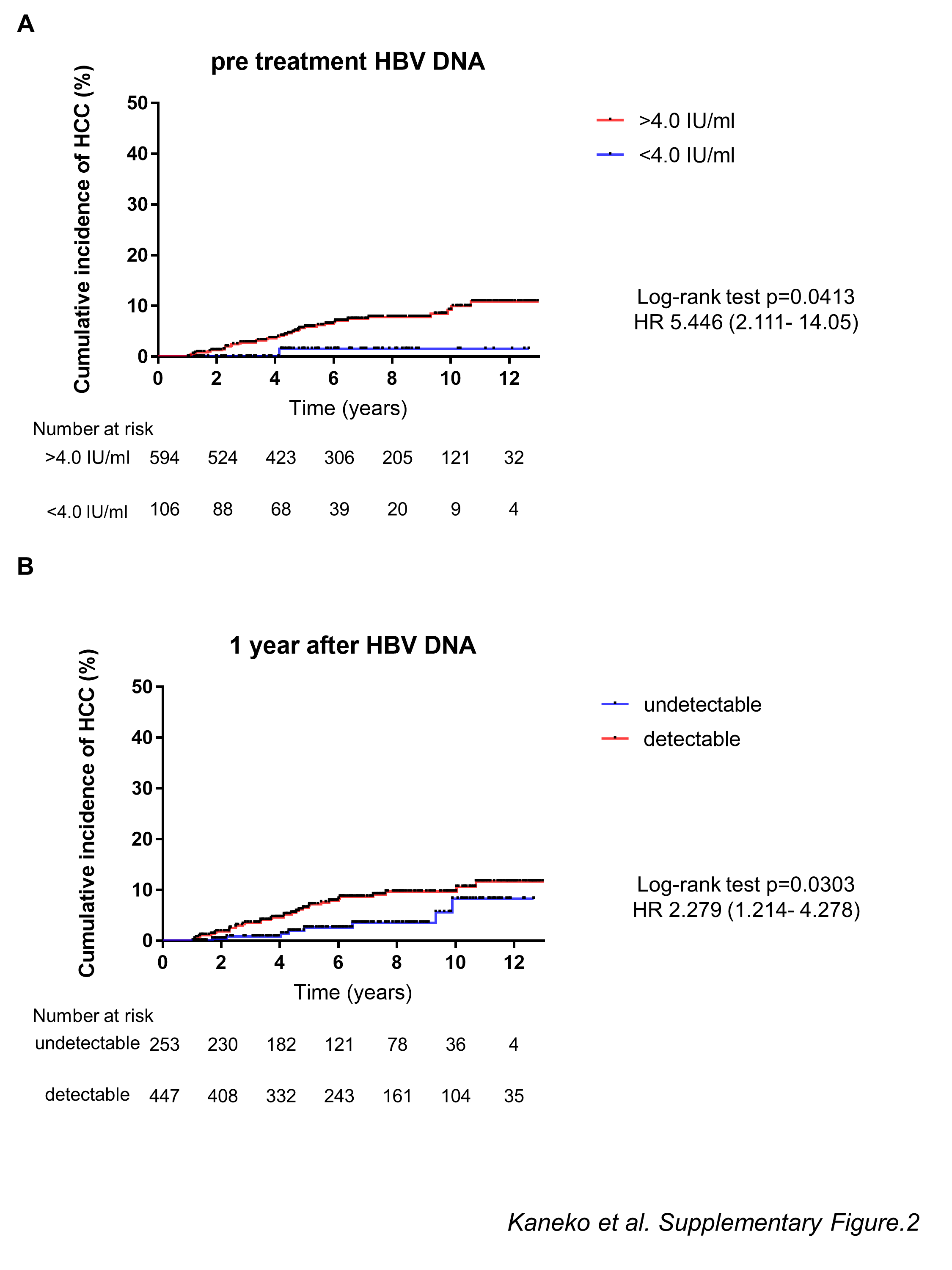

Supplement: Supplementary file 4 — Supplementary Figure 2. [file 41598_2020_69522_MOESM4_ESM.tif]

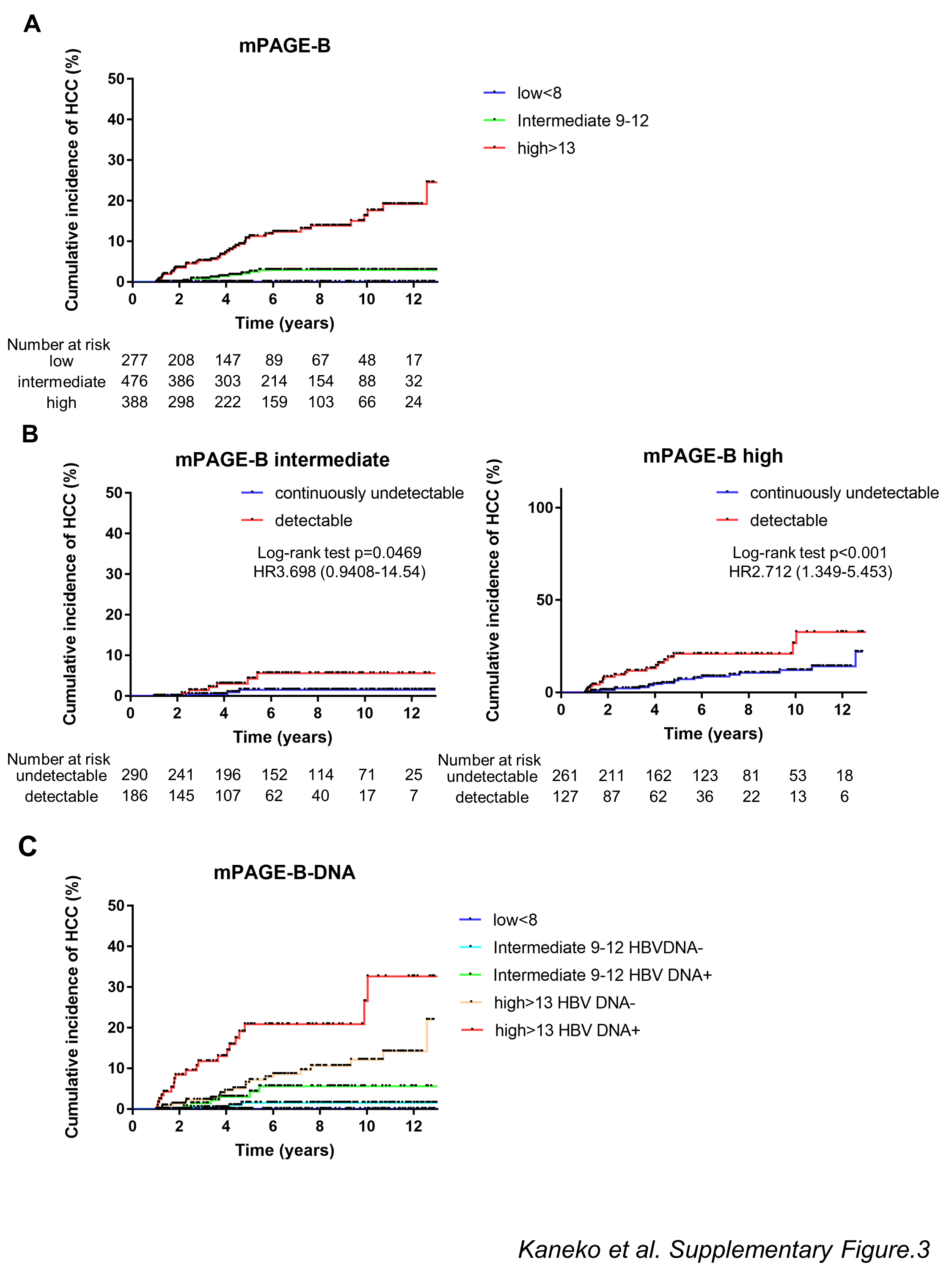

Supplement: Supplementary file 5 — Supplementary Figure 3. [file 41598_2020_69522_MOESM5_ESM.tif]
